# Increased Toll-Like Receptors Activity and TLR Ligands in Patients with Autoimmune Thyroid Diseases

**DOI:** 10.3389/fimmu.2016.00578

**Published:** 2016-12-09

**Authors:** Shiqiao Peng, Chenyan Li, Xinyi Wang, Xin Liu, Cheng Han, Ting Jin, Shanshan Liu, Xiaowen Zhang, Hanyi Zhang, Xue He, Xiaochen Xie, Xiaohui Yu, Chuyuan Wang, Ling Shan, Chenling Fan, Zhongyan Shan, Weiping Teng

**Affiliations:** ^1^Department of Endocrinology and Metabolism, Institute of Endocrinology, Liaoning Provincial Key Laboratory of Endocrine Diseases, The First Affiliated Hospital of China Medical University, China Medical University, Shenyang, Liaoning, China; ^2^Department of Endocrinology and Metabolism, The First Hospital of China Medical University, Shenyang, China; ^3^Department of Laboratory Medicine, The First Hospital of China Medical University, Shenyang, China; ^4^Department of Intensive Care Unit, Affiliated Hospital of Qingdao University, Qingdao, China; ^5^Department of Endocrinology, Sir Run Run Shaw Hospital, Affiliated to School of Medicine, Zhejiang University, Hangzhou, China; ^6^Department of Emergency, People’s Liberation Army No. 202 Hospital, Shenyang, China

**Keywords:** autoimmune thyroid disease, innate immunity, toll-like receptor, signaling, pathogenesis

## Abstract

**Objective:**

Autoimmune thyroid disease (AITD) is an organ-specific disorder due to the interplay between environmental and genetic factors. Toll-like receptors (TLRs) are pattern recognition receptors expressed abundantly on monocytes. There is a paucity of data on TLR expression in AITD. The aim of this study was to examine TLR expression, activation, ligands, and downstream signaling adaptors in peripheral blood mononuclear cells (PBMCs) extracted from untreated AITD patients and healthy controls.

**Method:**

We isolated PBMC of 30 healthy controls, 36 patients with untreated Hashimoto’s thyroiditis, and 30 patients with newly onset Graves’ disease. TLR mRNA, protein expression, TLR ligands, and TLR adaptor molecules were measured using real-time PCR, Western blot, flow cytometry, and enzyme-linked immunosorbent assay (ELISA). PBMC was simulated with TLR agonists. The effects of TLR agonists on the viability of human PBMC were evaluated using the MTT assay. The supernatants of cell cultures were measured for the pro-inflammatory cytokines, interleukin (IL)-6, tumor necrosis factor alpha (TNF-α), and IL-10 by ELISA.

**Results:**

TLR2, TLR3, TLR9, and TLR10 mRNA were significantly increased in AITD patients compared with controls. TLR2, TLR3, TLR9, high mobility group box 1 (HMGB1), and RAGE expression on monocytes was higher in patients than control at baseline and TLR agonists’ stimulation. The release of TNF-α and IL-6 was significantly increased in PBMCs from AITD patients with TLR agonists, while IL-10 was significantly decreased. Downstream targets of TLR, myeloid differentiation factor 88 (MyD88), and myeloid toll/IL-1 receptor-domain containing adaptor-inducing interferon-β were significantly elevated in AITD patients. Levels of TLR2 ligands, HMGB1, and heat shock protein 60 were significantly elevated in AITD patients compared with those in controls and positively correlated with TgAb and TPOAb, while sRAGE concentration was significantly decreased in AITD patients.

**Conclusion:**

This work is the first to show that TLR2, TLR3, and TLR9 expression and activation are elevated in the PBMCs of patients with AITD and TLRs may participate in the pathogenesis of AITD.

## Introduction

Autoimmune thyroid disease (AITD), which includes Hashimoto’s thyroiditis (HT) and Graves’ disease (GD) (1), is an organ-specific autoimmune condition that affects approximately 2–3% of the population in China. HT is characterized by substantial infiltration of thyroid-specific T and B lymphocytes and thyroid gland damage, eventually leading to hypothyroidism (2). GD is an autoimmune disorder characterized by the presence of autoantibodies that bind to and stimulate the thyroid stimulating hormone receptor, resulting in hyperthyroidism and goiter (3). Other than genetic and environmental factors, immune malfunction may also be involved in the development of AITD (4). Although the production of autoantibodies destroys self-tolerance and agitation of the adaptive immune system, the fundamental mechanism remains unclear.

Recent studies have demonstrated that aberrant activation of the innate immune response may have a significant effect on the pathogenesis of AITD. Due to an improved understanding of pattern recognition receptors, insight into innate immunity has expanded dramatically. Toll-like receptors (TLRs) are conserved pattern recognition receptors expressed on multiple types of cells, including monocytes, dendritic cells, B cells, and macrophages, and play a vital role in modulation of the innate immune system (5). TLRs recognize conserved molecules termed pathogen-associated molecular patterns (PAMPs), such as bacteria, viruses, and fungi, which act as ligands of specific TLR-induced signal transduction pathways (6), resulting in the activation of innate immune response and the release of inflammatory mediators, including interleukin (IL)-6, IL-12, IL-18, and tumor necrosis factor alpha (TNF-α). TLRs can also bind to certain endogenous molecules called damage-associated molecular patterns (DAMPs) and activate the innate immune response. Accordingly, TLRs have the ability to stimulate the production of monocytes and other antigen-presenting cells via activation of TLRs signaling pathways. To date, nothing is known regarding the ligands or cellular localization of TLR10, which is expressed only in humans (7). It is possible that TLR10 serves as a co-receptor for TLR2 (8). Ligands of TLR2 and TLR4 include heat shock protein 60 (HSP60), HSP70, hyaluronan, high mobility group box 1 (HMGB1), and advanced glycation end products (9). HSP60, the first endogenous ligand to be discovered, is an intramitochondrial chaperone that facilitates protein folding and stability (10). HSP60 also induce autoimmune responses by acting as a decoy to stimulate autoantibodies (11). HMGB1 has been described as a late inflammatory cytokine in several autoimmune diseases, such as rheumatoid arthritis (RA) (12) and systemic lupus erythematosus (SLE) (13). HMGB1 also plays a vital role in the initiation and progression of allograft rejection by acting as a “danger signal” (alarmin) (14). HMGB1 can interact with TLR2, TLR4, TLR9, and the receptor for advanced glycation end products (RAGE), thus inducing activation of TLR signaling. Once stimulated by ligands, TLRs recruit downstream adaptor molecules, including toll/IL-1 receptor-domain containing adaptor-inducing interferon-β (TRIF) and myeloid differentiation factor 88 (MyD88), resulting in signaling pathway activation and in the production of pro-inflammatory cytokines, such as IL-6 and TNF-α (15).

Toll-like receptors play an important role in the development of inflammatory disease. Previous studies have shown that TLRs are involved in the development of several autoimmune diseases, such as SLE, type 1 diabetes, and RA (16). TLR7 has been demonstrated to be involved in the production of autoantibodies and thus plays a vital role in the pathogenesis of SLE (17). TLR3, TLR7, and TLR9 are elevated in RA synovial fibroblasts (18), and a significant increase in TLR2 and TLR4 and their ligands has been described in T1DM patients (19, 20). In contrast, there is a paucity of studies regarding the role of TLR in AITD. Nonetheless, it has been demonstrated that TLR3 is highly expressed in thyrocytes from patients with HT (21), and increased expression of TLR3 was also observed in thyroid follicular epithelial cells from patients with GD (22). TLR3 signaling is activated by viral and bacterial molecules, dsRNA and poly (I:C), a synthetic viral analog, which results in the production of pro-inflammatory cytokines and interferons (IFNs) (22). It has also been demonstrated that TLR4 is expressed in FRTL-5 cells, leading to activation of NF-κB via recognition of LPS (23).

Although such findings based on animal and human thyroid gland investigations suggest that TLRs may play an important role in AITDs, it remains unclear whether activation of TLR pathways results in inflammation in AITD patients. The aim of the current study was to analyze TLR expression, activation, ligands, and downstream signaling adaptors in peripheral blood mononuclear cells (PBMCs) extracted from untreated AITD patients and healthy controls.

## Materials and Methods

### Study Groups

In this particular study, 4 mL fresh blood was obtained from 36 patients with HT, 30 patients with GD, and 30 age- and sex-matched controls. PBMCs were prepared from all samples. Serum samples were obtained from these patients. All patients were recruited from the First Affiliated Hospital of China Medical University. All participants provided a detailed history and underwent a complete physical examination as a condition of participation. None of the patients were taking evothyroxine or any other antithyroid drug. Subjects with any chronic disease, infectious disease, cancer, diabetes, or a family history of diabetes were excluded from this study. Levels of serum thyrotropin (TSH), free T3 (FT3), free T4 (FT4), TPOAb, TgAb, and thyrotrophin receptor antibody were analyzed using an electrochemiluminescent immunoassay with Cobas Elesys 601 (Roche Diagnostics Ltd., Switzerland). These samples were stored until use.

This experiment was approved by the Ethics Committee of the First Affiliated Hospital of China Medical University, and written informed consent was provided by each subject.

### Peripheral Blood Mononuclear Cell Isolation and TLR Agonist Stimulation

Peripheral blood mononuclear cells were obtained from freshly collected blood in heparinized tubes, isolated by Ficoll-isopaque density gradient centrifugation (Gibco BRL, Life Technologies Ltd., Paisley, UK), as described previously (24), and washed free of platelets and Ficoll. PBMCs were harvested and washed twice in RPMI 1640 (Gibco BRL, Life Technologies Ltd., Paisley, UK). The cells were diluted with complete medium consisting of RPMI 1640 supplemented with 10% fetal calf serum, 2 mmol/L glutamine, 100 U/mL penicillin G, and 100 U/mL streptomycin to 1 × 10^6^/mL. Cells were then dispensed in a 200-μL tube in 96-well plates. Pam3CSK4, Poly I:C, and CpG ODN were used as TLR2, TLR3, and TLR9 agonists, respectively. Each volume containing 1 × 10^6^ isolated PBMCs were incubated with medium, 1 μg/mL Pam3CSK4, 10 μg/mL poly I:C, and 1 μg/mL CpG ODN for 2, 6, 8, 12, 24 and 48 h at 37°C in 5% CO_2_. The supernatants were harvested and collected at −20°C until further use.

### Quantitative mRNA Expression of TLRs in PBMCs

RNA was extracted from PBMC, using TRIzol (Invitrogen Corp., CA, USA). The first strand of cDNA was synthesized using total RNA. RT-PCR was performed using TagMan gene expression assays, with glyceraldehydes-3-phosphatedehydrogenase (GAPDH) as a control (R&D Systems, Minneapolis, MN, USA). The sequences of primers for TLR1-10 and GAPDH were designed using the ABI PRISM system (Applied Biosystems). Sequences of the human primers for polymerase chain reaction were as summarized in Table 1. The primers were examined with BLAST software against the National Center for Biotechnology Information database. All PCRs were performed in a total volume of 20 μL, and single transcript of genes expression was determined using SYBR green master mix. Relative expression of TLR1-10 was calculated using the comparative cycle threshold method. TLR1-10 mRNA was expressed as a ratio to GAPDH.

**Table 1:** **Sequence of primers used to amplify**.

Genes	Forward primer	Reverse primer
TLR1	GGATGCAGAAGGAGATCACTG	TTTCAAAAACCGTGTCTGTTAAGAGA
TLR2	GGAGGCTGCATATTCCAAGG	GCCAGGCATCCTCACAGG
TLR3	CAGTGTCTGGTACACGCATGGA	TTTCAAAAACCGTGTCTGTTAAGAGT
TLR4	AGTTTCCTGCAATGGATCAAGG	CTGCTTATCTGAAGGTGTTGCA
TLR5	GGCTTAATCACACCAATGTCACTATAG	TTAAGACTTCCTCTTCATCACAACCTT
TLR6	CATCCTATTGTGAGTTTCAGGCAT	GCTTCATAGCACTCAATCCCAA
TLR7	TGGAAATTGCCCTCGTTGTT	GTCAGCGCATCAAAAGCATT
TLR8	AGCGGATCTGTAAGAGCTCCATC	CCGTGAATCATTTTCAGTCAAGAC
TLR9	AGCGGATCTGTAAGAGCTCCATC	CCGTGAATCATTTTCAGTCAAGA
TLR10	AAGAAAGGTTCCCGCAGACTT	TGTTATGGCATAGAATCAAAACTCTCA
GAPDH	CAA AGACCTGTACGCCAACA	GAAGCATTTGCGGTGGAC

### *In Vitro* Studies on PBMC from AITD Patients and Controls Cell Viability Assay

The effect of TLR agonists on the viability of human PBMC was analyzed by the MTT [3-(4,5-dimethylthiazol-2-yl)-2,5-diphenyltetra-zolium bromide] assay. PBMC were maintained in RPMI-1640 medium, supplemented with 10% fetal bovine serum, and 1% penicillin/streptomycin, under 37°C, 95% humidified air and 5% CO2. MTT was added to each well to a final concentration of 10 mg/L and the cells were continuously incubated for 5 h. After centrifugation for 5 min at 3,000 × *g*, the supernatant was removed and the wells were washed three times with PBS before addition of 100 mL isopropyl alcohol to dissolve MTT crystals and optical density (OD) was determined at 550 nm using micro enzyme-linked immunosorbent assay (ELISA) plate reader (Bio-Rad, Hercules, CA, USA). All measurements were performed in triplicate. Stimulation index was calculated as mean ratio of OD of the stimulated cells divided by OD of untreated cells.

### TLR2, TLR3, TLR9, HMGB1, and RAGE Expression in the Resting State and upon Specific Agonist Activation

Peripheral blood mononuclear cells were analyzed under resting and after 24 h stimulation with TLR2, 3, or 9 agonists. PBMCs were resuspended in PBS and stained for surface mAbs CD14-FITC (eBioscience, San Diego, CA, USA) and TLR2-APC (eBioscience, San Diego, CA, USA). After 15 min of incubation at room temperature in the dark, cells were washed and fixed in 1% paraformaldeyde in PBS. Cells were then permeabilized with saponin 0.5% (Sigma-Aldrich, St. Louis, MO, USA) and mAbs for TLR3-APC and TLR9-APC, with unconjugated mouse anti-human HMGB1 monoclonal antibody (Abcam, Cambridge, UK), unconjugated mouse anti-human RAGE polyclonal antibody (Millipore, MA, USA) or its corresponding mouse IgG isotype control (BD Biosciences Corp., San Jose, CA, USA) with subsequence incubation with APC-conjugated goat anti-mouse secondary antibody (Santa Cruz Biotechnology, CA, USA). Cells were incubated for 45 min at 4°C in the dark, washed, and fixed in 1% paraformaldeyde in PBS. Samples were then analyzed using BD FACS Array (Becton-Dickinson, Fullerton, CA, USA) equipped with software Cellquest.

Results are expressed as percentages of CD14^+^ TLR2^+^, CD14^+^ TLR3^+^, CD14^+^ TLR9^+^ cells, CD14^+^ HMGB1^+^, and CD14^+^ RAGE^+^. The intra- and inter-assay CVs for TLR2, TLR3, TLR9, HMGB1, and RAGE expression were found to be <5, <5, <15, <5, <10, and <15%, respectively.

### Cytokine Production

Interleukin-6, IL-10, and TNF-α were examined in the supernatants of the cultured PBMCs in AITD patients and healthy subjects after TLR agonist stimulation using commercially available ELISA kits (R&D, Minneapolis, MN, USA) according to the manufacturer’s instructions. Absorbance was measured at a wavelength of 450 nm using a microplate reader to analyze the intensity of color development in each well. The levels of cytokines were presented as picogram per milliliter.

### Western Blot Analysis

Western blot was used to detect protein in PBMC of AITD patients and controls. Frozen cells were homogenized in PBS (pH 7.4) supplemented with 0.05% Triton X-100 and protease inhibitor cocktail (Sigma). The protein concentration was determined using the Bradford method with BSA as the standard. SDS-PAGE was performed using 30% acrylamide (Sigma). After electrophoresis, gels were equilibrated for 20 min in transfer buffer (25 mM Tris, 190 mM glycine, and 20% methanol). Proteins were transferred onto polyvinylidene difluoride membranes (0.5 h, 30 V), which were then incubated with a blocking solution [5% dried skim milk in 100 mM Tris (pH 7.5) with 140 mM NaCl and 0.01% Tween 20 for a minimum of 1.5 h]. The blots were then incubated overnight at 4°C, either with a polyclonal rabbit anti-TRIF antibody (1:800, Abcam, UK), a polyclonal rabbit anti-MyD88 antibody (1:800, Abcam, UK), or a polyclonal rabbit anti-β-actin (1:1,000) on the same membrane. The blots were washed three times with the blocking solution and incubated with diluted horseradish-peroxidase-conjugated secondary antibodies (1:1,000) (Bio-Rad) for 1.0 h at room temperature. Blots were washed extensively and developed using an enhanced chemiluminescence kit (Amersham Pharmacia Biotech, Piscataway, NJ, USA). Western immunoblot bands were quantified by means of a Bio-Rad calibrated densitometer (GS-800) using the vendor’s software (Bio-Rad Laboratories) and β-actin served as an internal control for all analyses.

### Serum Levels of HMGB1, HSP60, and sRAGE

Serum levels of HMGB1, HSP60, and sRAGE were measured using commercially available ELISA kits (R&D, Minneapolis, MN, USA). The procedures were performed in accordance with the manufacturer’s instructions. Absorbance was measured at a wavelength of 450 nm using a microplate reader to analyze the intensity of color development in each well. The levels of HMGB1 and HSP60 were presented as nanograms per milliliter, whereas sRAGE was presented as picograms per milliliter.

### Statistical Analysis

All statistical analyses were performed using SPSS Software 20.0 (SPSS, Inc., Chicago, IL, USA). Results are displayed as mean ± SD. The normally distributed data were analyzed by independent-samples *t*-test. Meanwhile, the analysis of abnormally distributed data was performed with Kruskal–Wallis test followed by the Mann–Whitney *U* test. One-way ANOVA was used to compare three experimental groups. The correlation between serum levels and clinical variables was analyzed using the Spearman’s method. *P-*values <0.05 were considered statistically significant.

## Results

### Elevated Expression of TLR2, TLR3, TLR9, and TLR10 in PBMCs of AITD Patients

**Figure 1:**
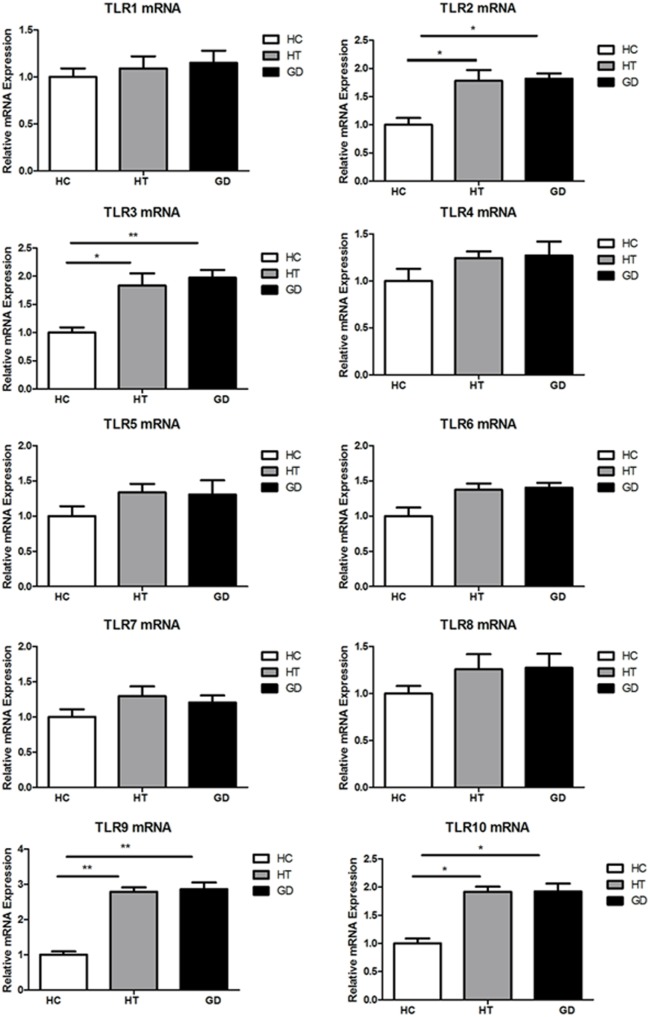
**Toll-like receptor (TLR) mRNA expression in peripheral blood mononuclear cells (PBMCs)**. Bar graphs comparing TLR1–TLR10 mRNA expression levels in PBMCs from patient with Hashimoto’s thyroiditis, Graves’s disease, and healthy controls. TLRs expression was measured by real-time PCR using GAPDH as endogenous control. Results are shown as fold change regarding TLRs gene expression in AITD patients relative to healthy subjects. Each bar represents the 1 ± SEM of three groups. One-way ANOVA was used to compare the differences of expression between AITD patients and healthy controls. AITD, autoimmune thyroid disease; HT, Hashimoto’s thyroiditis; GD, Graves’ disease; HC, healthy controls. Level of significant is *P < 0.05 and **P < 0.01.

Blood samples were obtained in 66 AITD patients and 30 healthy controls. The clinical features of enrolled subjects are shown in Table 2. In this study, the levels of expression of TLR1–TLR10 in PBMC samples from AITD patients (30 with untreated GD and 36 with HT) and 30 healthy subjects were assessed by RT-PCR were examined. As shown in Figure 1, the mRNA expression of TLR1, 4, 5, 6, 7, and 8 was equivalent between in AITD patients and healthy controls. The expression of TLR2, TLR3, TLR9, and TLR10 were significantly increased in both with HT (*P* < 0.05, *P* < 0.05, *P* < 0.01, and *P* < 0.05, respectively) and those with GD subjects (*P* < 0.05, *P* < 0.01, *P* < 0.01, and *P* < 0.05, respectively) than in controls, but no difference was found between HT and GD patients for all of these TLR.

**Table 2:** **The clinical characteristics of patients with Hashimoto’s thyroiditis, Graves’ disease, and healthy controls**.

Variable	HC	HT	GD	Normal range
No.	30	36	30	–
Age (years)	37 ± 8	34 ± 9	30 ± 9	–
Gender (M/F)	4/26	4/32	5/25	–
Thyrotropin (mIU/L)	1.3 ± 0.2	14.2 ± 3.16	0.007 ± 0.01	0.35–4.94
FT4 (ng/dL)	1.4 ± 0.1	0.9 ± 0.2	46.02 ± 14.7	9.01–19.05
FT3 (pg/mL)	2.6 ± 0.5	3.7 ± 0.2	34.3 ± 6.9	2.63–5.70
Thyrotrophin receptor antibody (IU/L)	–	–	37.6 ± 9.5	0–1
TPOAb (IU/mL)	5.03 ± 0.7	343.27 ± 126.2	167.5 ± 23.2	0.11–5.23
TgAb (IU/mL)	2.54 ± 0.5	433.45 ± 102.7	192.28 ± 17.32	0.81–3.83

*Data are expressed as mean ± SD. The “–” represents that the experiment was not performed, or the data are not applicable*.

*M, male; F, female; HT, Hashimoto’s thyroiditis; GD, Graves’ disease; HC, healthy controls*.

### Proliferative Response to TLR Agonists from PBMCs with AITD and HC

**Figure 2:**
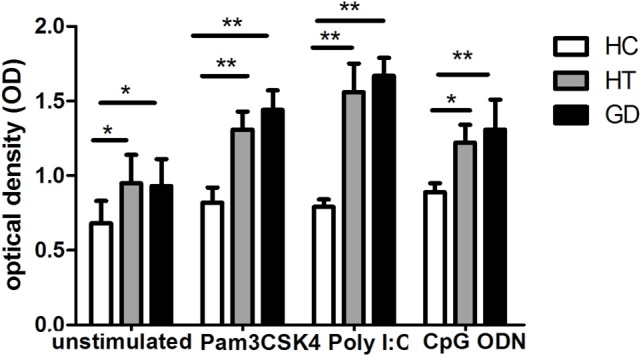
**Cell viability in peripheral blood mononuclear cells after toll-like receptor (TLR) agonists stimulation**. Bar graphs comparing of cell viability optical density between unstimulated and stimulated cells treated with either unstimulate or stimulate of TLR agonists in both AITD patients and controls by MTT assay. Each bar represents the mean ± SD of three groups. One-way ANOVA was used to compare the differences of expression between AITD patients and healthy controls. AITD, autoimmune thyroid disease; HT, Hashimoto’s thyroiditis; GD, Graves’ disease; HC, healthy controls. Pam3CSK4, Poly I:C, and CpG ODN were used as TLR2, TLR3, and TLR9 agonists, respectively. Level of significant is *P < 0.05 and **P < 0.01.

As shown in Figure 2, we found that PBMC from AITD patients significantly proliferated compared to controls (*P* < 0.05 for HT and GD, respectively). Increased OD was also found in AITD patients’ *in vitro* stimulation with TLR2 agonist (*P* < 0.01 for HT and GD, respectively). Similarly, there are increased OD in AITD patients when cultured with TLR3 and TLR9 agonists (*P* < 0.01 and *P* < 0.01 for Poly I:C and *P* < 0.05 and *P* < 0.01 for CpG ODN, respectively).

### Time-Dependent Modulation of TLR2, TLR3, TLR9, HMGB1, and RAGE Expression in PBMCs by Specific Agonist Activation

**Figure 3:**
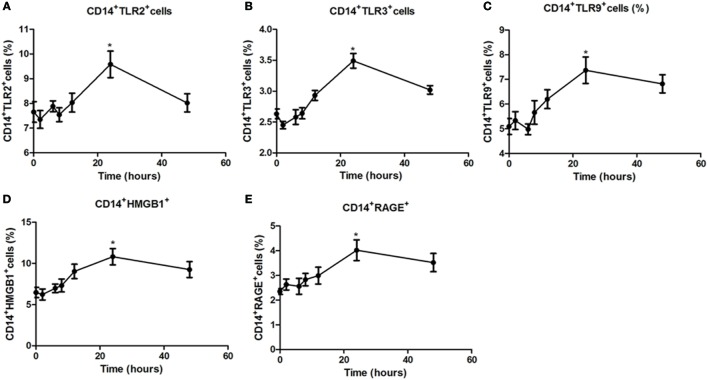
**Time-dependent modulation of TLR2, TLR3, TLR9, high mobility group box 1 (HMGB1), and receptor for advanced glycation end products (RAGE) expression in peripheral blood mononuclear cells (PBMCs) by specific agonist activation**. PBMCs from healthy controls were treated with a TLR2, TLR3, or TLR9 agonist for 0, 2, 6, 8, 12, 24, and 48 h stained with anti-CD14 and anti-TLR2, TLR3, TLR9, HMGB1, and RAGE mAbs for flow cytometry. Percentages of CD14^+^TLR2^+^ **(A)**, CD14^+^TLR3^+^ **(B)**, CD14^+^TLR9^+^ **(C)**, CD14^+^HMGB1^+^ **(D)**, CD14^+^RAGE^+^cells **(E)** from HC was measured by flow cytometry. Results are shown as mean ± SD of control group. Level of significant is *P < 0.05. Statistical analyses were conducted using one-way ANOVA. Pam3CSK4, Poly I:C, and CpG ODN were used as TLR2, TLR3, and TLR9 agonists, respectively.

To further characterize regulation of TLR expression by specific agonists, a time–response study was conducted. In PBMCs from healthy controls stimulated with specific TLR agonists for 2, 6, 8, 12, 24, and 48 h, the maximal response for TLR2, TLR3, TLR9, HMGB1, and RAGE protein expression was all observed at 24 h (all *P* < 0.05, Figure 3), and the expression significant reduced at 48 h, suggesting that 24 h is the optimal time for the PBMCs by TLR agonists stimulation.

### Responses of CD14^+^ Cells from AITD and HC to TLR2, TLR3, and TLR9 Stimulation with Pam3CSK4, Poly I:C, and CpG ODN

**Figure 4:**
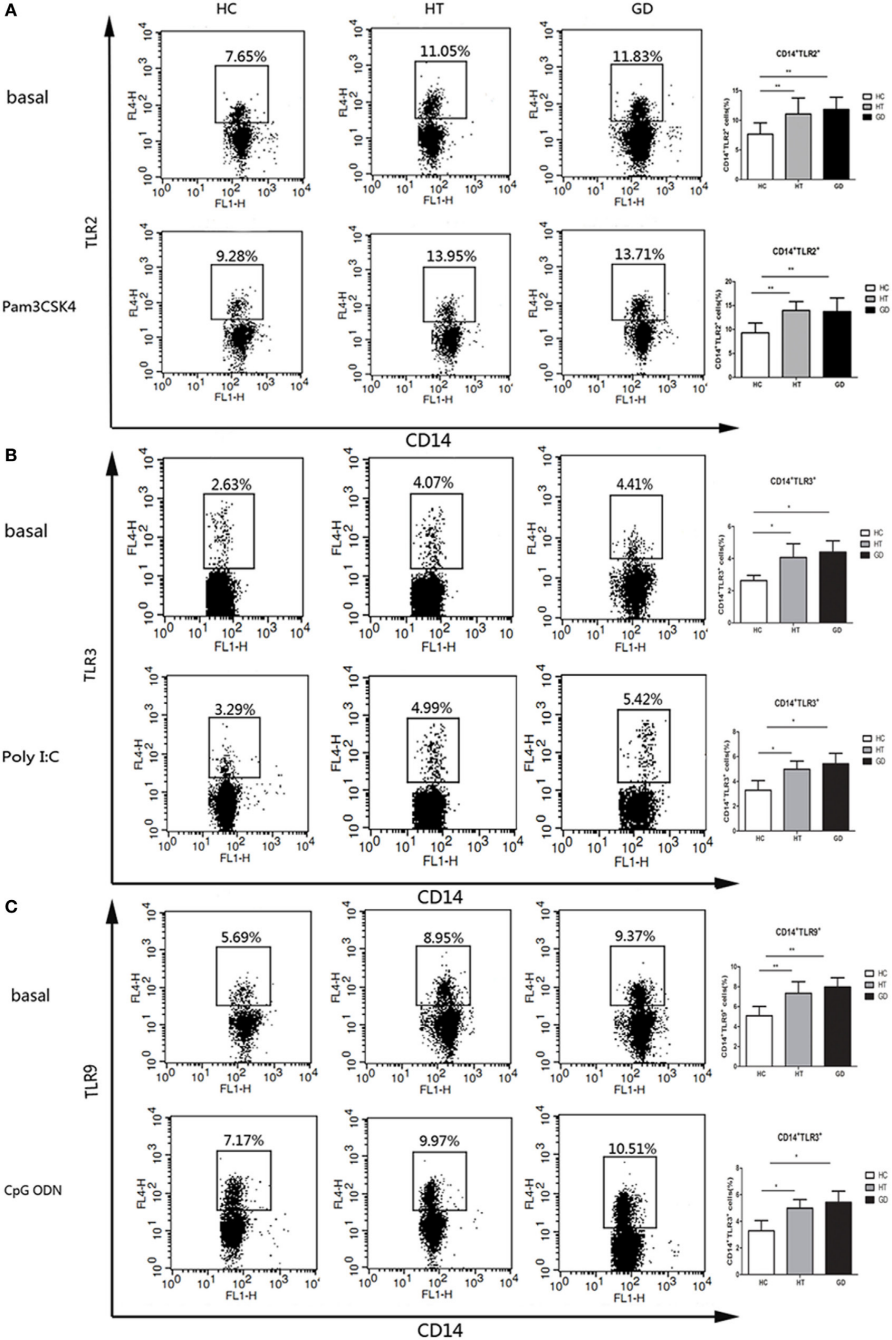
**TLR2, TLR3, and TLR9 surface protein expression in monocytes after toll-like receptor agonists stimulation**. Peripheral blood mononuclear cells from AITD patients and healthy controls were treated with a TLR2, TLR3, or TLR9 agonist or PBS as basal for 24 h and stained with anti-CD14 and anti-TLR2, TLR3, and TLR9 mAbs for flow cytometry. We first selected singlets using forward scatter area Vs forward scatter height parameters. From the singlet population, we removed dead cells from total events by gating on LIVE/DEAD events. Then, we selected total monocytes from the living using the pan-monocyte marker CD14, then TLR2 positive, TLR3 positive, and TLR9 positive, respectively. **(A)** Flow cytometry dot plots from AITD and HC depicting TLR2 expression by CD14 enriched monocyte cells (left panels). The number in the right quadrants represents the percentage of CD14^+^TLR2^+^ between AITD and healthy controls. **(B)** Flow cytometry dot plots from AITD and HC depicting TLR3 expression by CD14 enriched monocyte cells (left panels). Percentages of CD14^+^TLR3^+^ cells were compared between HC and AITD patients (right panels). **(C)** Flow cytometry dot plots from AITD and HC depicting TLR9 expression by CD14 enriched monocyte cells (left panels). Percentages of CD14^+^TLR9^+^ cells were compared between HC and AITD patients (right panels). Each bar represents the mean ± SD of three groups. Level of significant is *P < 0.05 and **P < 0.01. AITD, autoimmune thyroid disease; HT, Hashimoto’s thyroiditis; GD, Graves’ disease; HC, healthy controls. Statistical analyses were conducted using one-way ANOVA. Pam3CSK4, Poly I:C, and CpG ODN were used as TLR2, TLR3, and TLR9 agonists, respectively.

Intracellular and surface expression upon TLR stimulation in CD14^+^ cells were analyzed in PBMCs from AITD and control subjects. The expression of TLR2 was analyzed in HT, GD, and HC groups of CD14^+^ monocytes cells after cultured for 24 h in the presence or absence of Pam3CSK4. As shown in Figure 4A, the percentage of CD14^+^ TLR2 monocytes was significantly higher in AITD patients than in controls in resting and Pam3CSK4 activation (*P* < 0.01 and *P* < 0.01, respectively). Similarly, in both resting and agonist-stimulated cultured cells, TLR3 and TLR9 expression on peripheral CD14^+^ monocytes expression in AITD patients was significantly higher than in the control group (*P* < 0.05 and *P* < 0.05; *P* < 0.01 and *P* < 0.01 respectively, Figures 4B,C). TLR2, TLR3, and TLR9 expression did not differ between GD and HT patients (Table 3).

**Table 3:** **Percentage of TLR2, TLR3, and TLR9 expressing CD14^+^ cells in basal condition and after stimulation of AITD and HC PBMCs with Pam3CSK4 (TLR2), Poly I:C (TLR3), and CpG ODN (TLR9) for 24 h**.

	Basal			Pam3CSK4	Poly I:C	CpG ODN
	TLR2	TLR3	TLR9	TLR2	TLR3	TLR9
HC	7.6 ± 2.0	2.6 ± 0.3	5.1 ± 1.0	9.3 ± 2.7	3.2 ± 0.7	6.6 ± 1.2
HT	11.8 ± 2.1	4.1 ± 0.7	7.4 ± 1.2	13.8 ± 1.8	5.0 ± 0.6	9.8 ± 2.0
GD	11.0 ± 2.7	4.4 ± 0.8	7.9 ± 0.9	13.5 ± 2.9	5.3 ± 0.8	10.4 ± 1.9

*Results are shown as mean ± SD of three groups*.

*AITD, autoimmune thyroid disease; HT, Hashimoto’s thyroiditis; GD, Graves’ disease; HC, healthy controls*.

*Pam3CSK4, Poly I:C, and CpG ODN were used as TLR2, TLR3, and TLR9 agonists, respectively*.

### Responses of CD14^+^ Cells from AITD and HC to HMGB1 and RAGE Stimulation with CpG ODN

**Figure 5:**
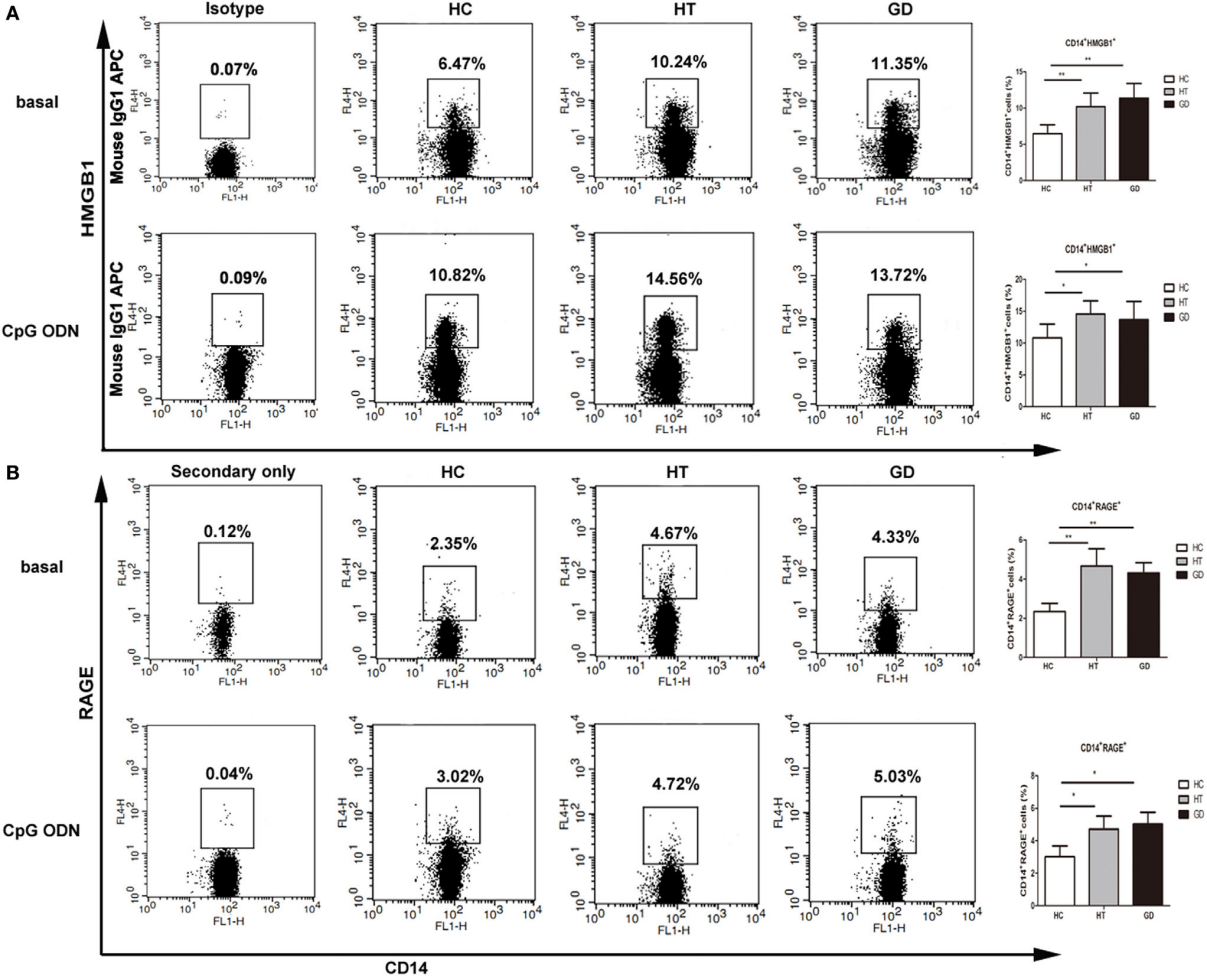
**High mobility group box 1 (HMGB1) and receptor for advanced glycation end products (RAGE) surface protein expression in monocytes after TLR9 agonist stimulation**. Peripheral blood mononuclear cells from AITD patients and healthy controls were treated with a TLR9 agonist or PBS as basal for 24 h and stained with anti-CD14 and anti-HMGB1, and RAGE mAbs for flow cytometry. We first selected singlets using forward scatter area Vs forward scatter height parameters. From the singlet population, we removed dead cells from total events by gating on LIVE/DEAD events. Then, we selected total monocytes from the living using the pan-monocyte marker CD14, then HMGB1 positive and RAGE positive cells selected, respectively. **(A)** Flow cytometry dot plots from AITD and HC depicting HMGB1 or match isotype control expression by CD14 enriched monocyte cells (left panels). The number in the right quadrants represents the percentage of CD14^+^HMGB1^+^ between AITD and healthy controls. **(B)** Flow cytometry dot plots from AITD and HC depicting RAGE expression by CD14 enriched monocyte cells (left panels). Percentages of CD14^+^RAGE^+^ cells were compared between HC and AITD patients (right panels). Control cells were stained with the secondary antibodies without using the anti-RAGE primary antibody. Each bar represents the mean ± SD of three groups. Level of significant is *P < 0.05 and **P < 0.01. AITD, autoimmune thyroid disease; HT, Hashimoto’s thyroiditis; GD, Graves’ disease; HC, healthy controls. Statistical analyses were conducted using one-way ANOVA. CpG ODN was used as a TLR9 agonist.

The expression of HMGB1 and RAGE were analyzed in HT, GD, and HC groups of CD14^+^ monocytes cells when cultured with TLR9 agonist, CpG ODN. As shown in Figure 5A, the percentage of CD14^+^ HMGB1 monocytes was significantly higher in AITD patients than in controls in resting (11.3 ± 2.0 for HT, 10.6 ± 1.8 for GD Vs 6.5 ± 1.2 for HC) and CpG ODN activation (14.7 ± 2.2 for HT, 13.8 ± 2.6 for GD Vs 10.5 ± 2.1 for HC; all *P* < 0.01, respectively). Similarly, the percentage of CD14^+^ RAGE^+^ cells in AITD patients was significantly higher than those in control group in both resting (4.7 ± 0.7 for HT, 4.3 ± 0.5 for GD Vs 2.3 ± 0.4 for HC) and agonist-stimulated cultured cells (4.9 ± 0.8 for HT, 5.0 ± 0.7 for GD Vs 3.0 ± 0.5 for HC; *P* < 0.01 for HT and *P* < 0.01 for GD, respectively, Figure 5B).

### The Expression of TNF-α, IL-6, and IL-10 in PBMCs Stimulated with TLR2, TLR3, and TLR9 Agonists Pam3CSK4, Poly I:C, and CpG ODN

**Figure 6:**
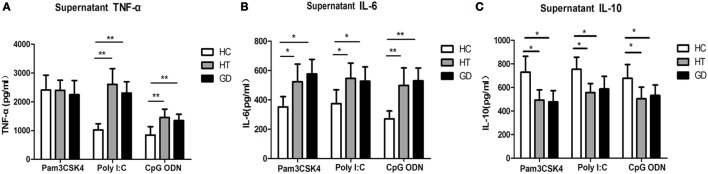
**Release of tumor necrosis factor alpha (TNF-α), interleukin (IL)-6, and IL-10 in the cultured supernatants from AITD patients and healthy controls**. **(A)** Peripheral blood mononuclear cells (PBMCs) isolated from subjects were stimulated with Pam3CSK4, poly I:C, and CpG ODN for 24 h, TNF-α were measured by enzyme-linked immunosorbent assay (ELISA). **(B)** PBMCs were treated as in **(A)** and the release of IL-6 was measured by ELISA. **(C)** PBMCs were treated as in **(A)** and the release of IL-10 was measured by ELISA. Level of significant is *P < 0.05 and **P < 0.01. Values were shown as mean ± SD and expressed as picograms per milliliter. AITD, autoimmune thyroid disease; HT, Hashimoto’s thyroiditis; GD, Graves’ disease; HC, healthy controls. Statistical analyses were conducted using one-way ANOVA. Pam3CSK4, poly I:C, and CpG ODN were used as TLR2, TLR3, and TLR9 agonists, respectively.

To determine whether TLR activation can induce cytokine upregulation in AITD patients, we examine the cytokine in TLR agonist activation PBMCs from AITD patients and healthy controls. TNF-α, IL-6, and IL-10 expression were evaluated in the culture supernatant of PBMCs after stimulation with Pam3CSK4, Poly I:C, and CpG ODN for 24 h. As shown in Figure 6A, the TNF-α expression in AITD patients, including GD and HT patients, were found to be significantly higher than those for control subjects when stimulated with either Poly I:C (TLR3) or CpG ODN (TLR9) (*P* < 0.01 and *P* < 0.01 for Poly I:C and CpG ODN, respectively). However, the expression of TNF-α in response to Pam3CSK4, a ligand of TLR2, appeared to be the same in all three groups. As shown in Figure 6B, the IL-6 secretion levels from PBMC samples stimulated with either Pam3CSK4, Poly I:C, or CpG ODN in the AITD groups were also found to be significantly higher than those in the control group (*P* < 0.05, *P* < 0.05, and *P* < 0.01 for Pam3CSK4, Poly I:C, and CpG ODN, respectively), while IL-10 expression, were significantly decreased in AITD groups under stimulation (*P* < 0.01, *P* < 0.01, and *P* < 0.01 for Pam3CSK4, Poly I:C, and CpG ODN, respectively, Figure 6C).

### Downstream Proteins under Western Blot Analysis

**Figure 7:**
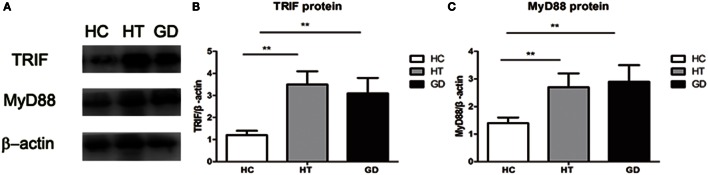
**Toll-like receptor (TLR) signaling proteins in peripheral blood mononuclear cell from patients with Hashimoto’s thyroiditis, Graves’s disease, and healthy controls**. **(A)** Representative Western blotting results of TLR downstream signaling proteins MyD88 and toll/IL-1 receptor-domain containing adaptor-inducing interferon-β (TRIF) was performed using specific antibodies to the respective proteins as described in methods. β-actin was used as loading and internal control for MyD88 and TRIF. **(B)** A ratio of TRIF/β-actin was determined to represent a mean net density. **(C)** A ratio of MyD88/β-actin was determined to represent a mean net density. Level of significant is *P < 0.05 and **P < 0.01. Values were shown as mean ± SD. HT, Hashimoto’s thyroiditis; GD, Graves’ disease; HC, healthy controls. One-way ANOVA was used to compare the differences of TRIF and MyD88 proteins of these three groups.

The downstream proteins TRIF and MyD88 were analyzed by Western blot analysis. As shown in the Figures 7A,B, the expression of TRIF was significantly higher in PBMC samples from HT and GD patients than in those from healthy subjects (*P* < 0.01 and *P* < 0.01, respectively). There was no significant difference between the HT and GD subjects.

MyD88 expression in HT and GD groups was also significantly higher than in HC group (*P* < 0.01; *P* < 0.01, respectively. Figures 7A,C). No significant difference was found between HT and GD groups.

### Elevated HMGB1, HSP60, and sRAGE in the Serum of AITD Patients

**Figure 8:**
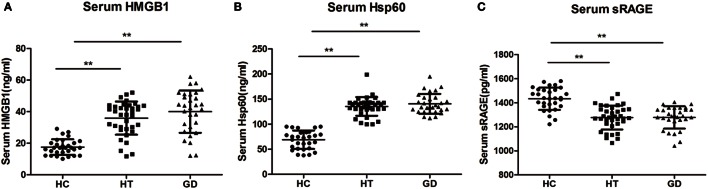
**Dot plots showing serum levels of high mobility group box 1 (HMGB1), heat shock protein 60 (HSP60), and sRAGE in patients with Hashimoto’s thyroiditis, Graves’s disease, and healthy controls**. Serum levels of HMGB1 **(A)**, HSP60 **(B)**, and sRAGE **(C)** were measured by using enzyme-linked immunosorbent assay. Results are shown in the graphic. HT, Hashimoto’s thyroiditis; GD, Graves’ disease; HC, healthy controls. Level of significant is *P < 0.05 and **P < 0.01. Values were shown as mean ± SD. Statistical analyses were conducted using one-way ANOVA.

The serum levels of HMGB1 were significantly higher in patients with HT (37.81 ± 12.26 ng/mL) and in patients with GD (40.03 ± 13.40 ng/mL) than in control subjects (17.54 ± 4.94 ng/mL, *P* < 0.01, and *P* < 0.01, respectively, Figure 8A). Similarly, the HSP60 levels were also higher in HT (135.33 ± 18.63 ng/mL) and GD groups (140.55 ± 19.81 ng/mL) than in control subjects (68.99 ± 18.34 ng/mL, *P* < 0.01, and *P* < 0.01, respectively, Figure 8B). In addition, the serum levels of HMGB1 and HSP60 did not differ between GD and HT patients. In contrast, there was a significantly decreased expression of sRAGE in HT (1,276.67 ± 98.47 pg/mL) and GD patients (1,278.08 ± 92.54 pg/mL) than in healthy subjects (1,433.80 ± 92.48 pg/mL, *P* < 0.01, and *P* < 0.01, respectively, Figure 8C).

### Correlation of HMGB1, HSP60, and sRAGE with Clinical Variables

**Figure 9:**
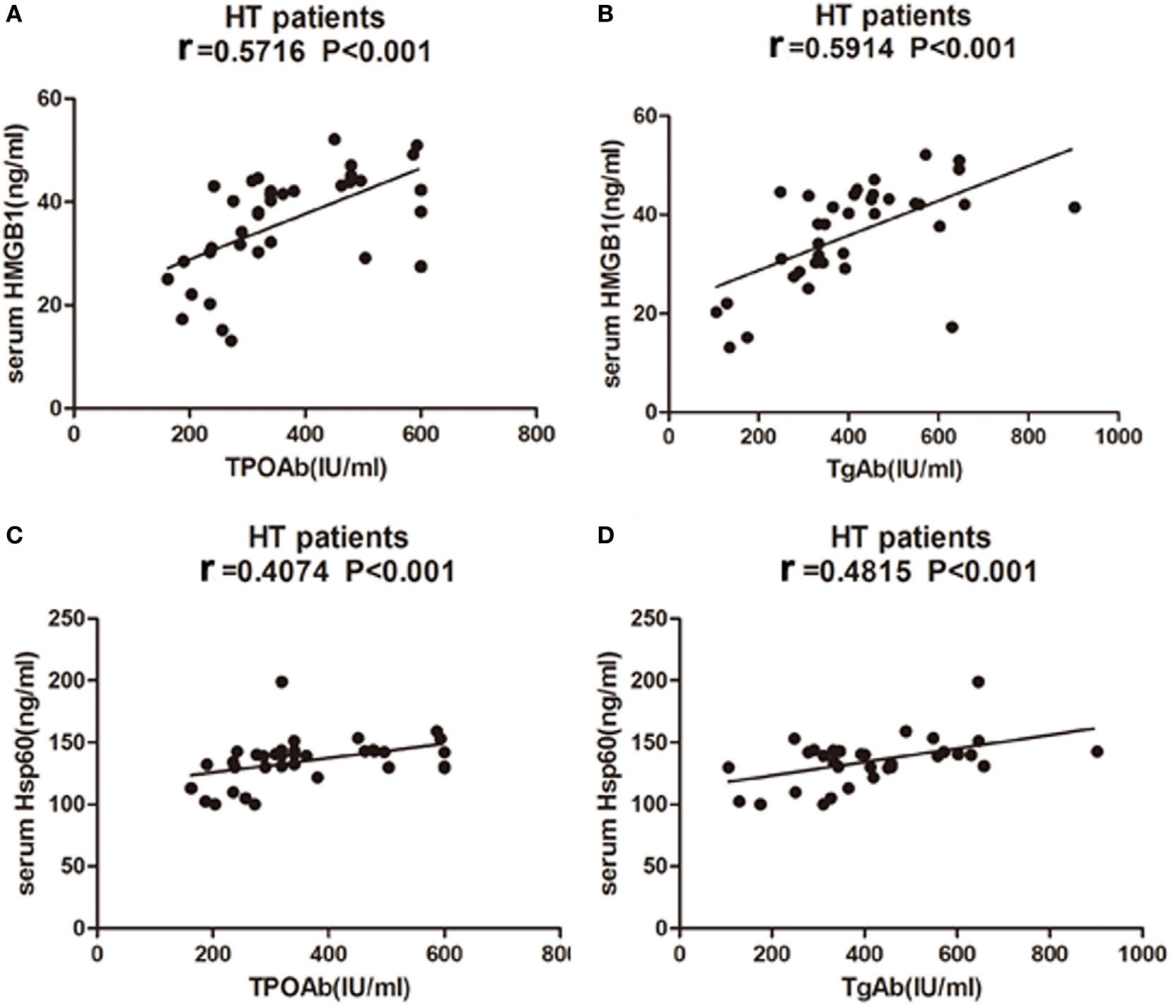
**Correlation analysis of laboratory parameters and serum levels of high mobility group box 1 (HMGB1) and heat shock protein 60 (HSP60) in HT patients**. Relationship between serum expression of HMGB1 with TPOAb **(A)** and TgAb **(B)**, HSP60 with TPOAb **(C)** and TgAb **(D)** are presented in scatter plots. Pearson’s and Spearman’s correlation and *P*-values are given in all cases, *r* = Pearson correlation coefficient. HT, Hashimoto’s thyroiditis.

No significant correlation was found between either HMGB1, HSP60, or sRAGE levels and serum levels of TSH, FT3, or FT4 in HT patients. However, HMGB1 showed a significant association with TPOAb and TgAb in HT patients (*r* = 0.5716, *P* < 0.001, Figure 9A; *r* = 0.5914 *P* < 0.001, Figure 9B). There was also a significant correlation between HSP60 and TPOAb (*r* = 0.4074, *P* < 0.001, Figure 9C) and TgAb (*r* = 0.4815 *P* < 0.001, Figure 9D) in HT patients. However, no significant correlations were found between sRAGE expression and clinical variables.

## Discussion

Autoimmune thyroid diseases are characterized by production of pathogenic thyroid autoantibodies and lymphocyte infiltration (1). TLRs play a vital role in the increased inflammation observed in AITD, which may be mediated in part through activation of innate immune pathways by TLRs. However, no study to date has examined TLR expression in PBMCs from AITD patients or their contribution to the production of pro-inflammatory cytokines.

In the current study, mRNA expression of TLR2, -3, -9, and -10 was significantly increased in patients with HT and GD compared with controls. Despite the lack of data regarding TLR in AITD, TLR3 has been found to be highly expressed by thyrocytes and thyroid follicular epithelial cells of HT and GD patients (21). TLR3 is also expressed in both thyroid follicles and inflammatory cells of iodine-induced NOD.H-2^h4^ mice (25), a classic model of spontaneous autoimmune thyroiditis (26). In addition, poly (I:C) administration aggravates inflammation of excessive iodine-induced thyroiditis in NOD mice, and evidence of the severity of thyroid follicle destruction and amount of lymphocyte infiltration has been reported (25). DNA microarray analysis has shown that stimulation of cultured human thyroid follicles with dsRNA [poly (I:C)] can cause activation of inflammatory responses and upregulation of a variety of related genes, including pro-inflammatory cytokines (IL-6) and chemokines (27). The results of the present study are consistent with previous studies suggesting that TLR3 may be involved in the development of AITD. In our study, mRNA expression of TLR2 was found to be elevated in AITD patients. It has been reported that FRTL-5 cells express TLRs, including TLR2, TLR3, TLR4, and TLR9, and stimulate the expression of pro-inflammatory cytokine (27). Thyrocytes produce pro-inflammatory cytokines and type I IFNs in response to PAMPs and DAMPs, thereby activating the innate immune response (28). Here, we present evidence that TLR9 was more highly expressed in PBMCs from HT and GD patients compared to controls. Although TLR9 has been demonstrated to be associated with SLE (29) and type 1 diabetes (30), there are limited data regarding the levels of TLR9 in AITD patients. A previous study demonstrated that TLR9 single-nucleotide polymorphisms (SNPs) play an important role in males with thyroid-associated ophthalmopathy (31). TLR9 recognizes the unmethylated CpG motif of microbial DNA and endogenous DNA, which acts as a PAMP and binds to TLR9 to stimulate signal transduction via the MyD88 pathway against viral pathogens. A recent study has shown a significant association between TLR10 SNPs and the mechanism of AITD in patients (32). TLR10 is expressed by immune-related cells and is more likely to interact with TLR1 and TLR6 than other TLRs. Although the ligands and the signaling of TLR10 remain unknown (7), it has been reported that TLR10 functions as a co-receptor of TLR2 (33). The underlying mechanism of TLR10 in AITD remains unknown. Further studies are needed to confirm the role of TLR10 in thyrocytes.

A remarkable increase in viability was found for PBMCs from AITD patients in the resting state and after TLR stimulation. This finding indicates that activation of TLR signaling triggers abnormal activation of lymphocytes and stimulates and amplifies the immune response in AITD patients, which may lead to persisting autoimmunity.

TLR2, TLR3, and TLR9 surface expression is increased on monocytes from AITD subjects compared with controls. In addition, following specific agonist stimulation, the percentage of TLR2-expressing CD14^+^ cells increased in the PBMCs of both HT and GD patients, suggesting further activation of signaling pathways. Similarly, increased TLR3 and TLR9 expression in AITD patients was also found under TLR3 and TLR9 stimulation. HMGB1 and RAGE expression was found to be upregulated in PBMCs from AITD patients in the resting state and under TLR9 stimulation. HMGB1, a nuclear protein released from damaged or necrotic cells, is involved in many chronic inflammatory diseases. The HMGB1–DNA complex binds to RAGE and then activates the TLR9-mediated inflammatory response (34), and a previous study showed that extracellular HMGB1 could bind to CpG ODN and stimulate its transfer to TLR9 in inflammatory disease (35). Our finding suggested that HMGB1 and RAGE play an important role in the development of chronic inflammation in AITD through the activation of TLR9 pathway.

Toll-like receptor activation has been demonstrated to have the ability to regulate the production of pro-inflammatory cytokines (36, 37). Our results showed increased IL-6 and decreased IL-10 production in PBMCs from AITD patients in response to specific ligands for either TLR2, TLR3, or TLR9 and increased TNF-α expression in response to TLR3 and TLR9 ligands compared to healthy controls. Conversely, TNF-α secretion after TLR2 agonist stimulation in PBMCs from AITD patients and controls was equivalent, suggesting that TNF-α production may not be connected to TLR2 activation in cultured PBMCs. This finding appears to be consistent with a previous study in gout patients, whereby TNF-α secretion after Pam3CSK4 stimulation by PBMCs from gout patients was not significantly different from controls (38). These findings indicate that PBMCs from patients with AITD may be more sensitive to response induced by the agonists of TLRs, resulting in the secretion of pro-inflammatory cytokines.

Toll-like receptors act mainly through the adaptor molecule MyD88, which results in transcription of the NF-κB gene and activation of pro-inflammatory cytokines, such as IL-6 and TNF-α (39, 40). TRIF is also important to the TLR3 and TLR4 signaling pathways. In the present study, we found increased expression of downstream adapter proteins MyD88 and TRIF in PBMCs from patients with AITD compared with controls. Increased mRNA expression of MyD88, TRAF6, and IRAK4 has been demonstrated in PBMCs from SLE patients, which correlated with disease activity (41). Increased expression of MyD88 in B lymphocytes was also found in SLE patients but unlikely to be affected by disease activity (42). Increased expression of MyD88, TRIF, and IRAK has been found in monocytes from patients with T1DM (20, 43). The results of these studies are consistent with our results, suggesting that MyD88-dependent and -independent pathways are activated in the development of AITD.

Expression of endogenous ligands (HSP60 and HMGB1) for TLR2 and TLR4 and sRAGE was also examined in this study. We found HSP60 and HMGB1 levels to be significantly increased in the serum of AITD patients compared with controls. Moreover, the serum levels of sRAGE were significantly decreased in AITD patients. A previous study has shown that HMGB1 is overexpressed in the thyroid of HT patients, and HMGB1 expression is very low in healthy thyroids (44). Recent results also revealed increased numbers of HMGB1-positive monocytes in GD patients compared to healthy controls; with expression decreased by approximately 50% under antithyroid drug treatment (45). HSP60 is an intracellular chaperone that plays an important role in protein folding and stability. Kotani et al. first demonstrated that HSP60 is more highly expressed in HT follicular cells than in normal thyrocytes (46), and it has been reported that the serum levels of HSP60 are significantly increased in HT patients compared to controls (47). HSP60 is expressed in the thyroid glands of HT subjects and is localized to thyrocytes. This previous study also reported a correlation between HSP60 and both TgAb and TPOAb antibodies in the serum of HT patients. These studies suggest that HSP60 may actively participate in the development of HT through antibody recognition. This is the first finding regarding the elevated HSP60 expression in GD patients. Although no studies have addressed the levels of serum HSP60 in GD patients, HSP70, another member of the HSP family, is highly expressed in the thyroid tissue of patients with GD or HT (48, 49).The results of the current study show significantly decreased serum levels of sRAGE in HT and GD patients compared with controls. It has recently been reported that levels of sRAGE are significantly decreased in HT patients and that serum levels of AGEs are inversely correlated with those of sRAGE, a finding that is consistent with the results of the current study. Another study also showed increased expression of AGEs in HT patients, with positive correlations with TPOAb (50). A tendency toward elevated expression of AGEs in hyperthyroidism patients has also been reported, but this was significant only in patients with GD, which is consistent with our results (51). The serum levels of HSP60 and HMGB1 tend to correlate with TgAb and TPOAb in HT patients. These findings suggest that HSP60 and HMGB1 may play a vital role in the development of HT and are thus related to disease progression.

## Conclusion

This is the first study reporting increased TLR expression, activation, and signaling adaptor molecules in PBMCs from AITD patients; a significant elevation in TLR endogenous ligands was also observed in the serum of AITD group. In a future study, the mechanism underlying the effects of viral and endogenous ligands on TLR activation and signaling pathways will be examined.

## Author Contributions

Conceived and designed the experiments: WT, SP, and CL. Performed the experiments: SP, CL, XL, CH, TJ, SL, XZ, XH, HZ, XX, and CF. Analyzed the data: XW, SP, and CL. Contributed reagents/materials/analysis tools: WT, LS, ZS, XY, and CW. Contributed to the writing of the manuscript: SP and CL. All the authors reviewed and approved the final manuscript.

## Conflict of Interest Statement

The authors declare that the research was conducted in the absence of any commercial or financial relationships that could be construed as a potential conflict of interest.
