# Corrigendum: Increased Toll-Like Receptors Activity and TLR Ligands in Patients with Autoimmune Thyroid Diseases

**DOI:** 10.3389/fimmu.2017.00551

**Published:** 2017-05-15

**Authors:** Shiqiao Peng, Chenyan Li, Xinyi Wang, Xin Liu, Cheng Han, Ting Jin, Shanshan Liu, Xiaowen Zhang, Hanyi Zhang, Xue He, Xiaochen Xie, Xiaohui Yu, Chuyuan Wang, Ling Shan, Chenling Fan, Zhongyan Shan, Weiping Teng

**Affiliations:** ^1^Department of Endocrinology and Metabolism, Institute of Endocrinology, Liaoning Provincial Key Laboratory of Endocrine Diseases, The First Affiliated Hospital of China Medical University, China Medical University, Shenyang, Liaoning, China; ^2^Department of Endocrinology and Metabolism, The First Hospital of China Medical University, Shenyang, China; ^3^Department of Laboratory Medicine, The First Hospital of China Medical University, Shenyang, China; ^4^Department of Intensive Care Unit, Affiliated Hospital of Qingdao University, Qingdao, China; ^5^Department of Endocrinology, Sir Run Run Shaw Hospital, Affiliated to School of Medicine, Zhejiang University, Hangzhou, China; ^6^Department of Emergency, People’s Liberation Army No.202 Hospital, Shenyang, China

**Keywords:** autoimmune thyroid disease, innate immunity, toll-like receptor, signaling, pathogenesis

There was a mistake in the address of the author’ unit 1 as published. The unit 1 in the original article was not comprehensive and did not include the affiliated hospital of China medical university. The correct address of the unit 1 should be “Department of Endocrinology and Metabolism, Institute of Endocrinology, Liaoning Provincial Key Laboratory of Endocrine Diseases, The First Affiliated Hospital of China Medical University, China Medical University, Shenyang, Liaoning, China.” The correct address of author’ unit 1 is below.

**Address**

Department of Endocrinology and Metabolism, Institute of Endocrinology, Liaoning Provincial Key Laboratory of Endocrine Diseases, The First Affiliated Hospital of China Medical University, China Medical University, Shenyang, Liaoning, China

The authors apologize for this oversight. This error does not change the scientific conclusions of the article in any way.

The original article has been updated.

## Conflict of Interest Statement

The authors declare that the research was conducted in the absence of any commercial or financial relationships that could be construed as a potential conflict of interest.

